# Nanotechnology in Pediatric Neurology: Applications and Innovations

**DOI:** 10.3390/pharmaceutics18080999

**Published:** 2026-08-13

**Authors:** Raluca Ioana Teleanu, Ioana Alexandra Lungescu, Adelina-Gabriela Niculescu, Ana Cojocaru, Radu Ștefan Perjoc, Bianca Teodora Chenescu, Eugenia Roza, Oana Aurelia Vladâcenco, Alexandru Mihai Grumezescu, Daniel Mihai Teleanu

**Affiliations:** 1 “Carol Davila” University of Medicine and Pharmacy, 020021 Bucharest, Romania; raluca.teleanu@umfcd.ro (R.I.T.); radu-stefan.perjoc@rez.umfcd.ro (R.Ș.P.); bianca-teodora.ciurez@rez.umfcd.ro (B.T.C.); eugenia.roza@umfcd.ro (E.R.); oana-aurelia.vladacenco@drd.umfcd.ro (O.A.V.); daniel.teleanu@umfcd.ro (D.M.T.); 2 National University of Science and Technology POLITEHNICA Bucharest, 011061 Bucharest, Romania; alexandralungescu@gmail.com (I.A.L.); adelina.niculescu@upb.ro (A.-G.N.); agrumezescu@upb.ro (A.M.G.); 3 Research Institute of the University of Bucharest—ICUB, University of Bucharest, 050657 Bucharest, Romania

**Keywords:** pediatric neurology, nanotechnology, smart nanocarriers, theranostics, pediatric pharmacokinetics, pediatric brain tumors, AI-driven nanomedicine, brain organoids, digital twins, clinical translation

## Abstract

Nanotechnology is rapidly transforming the perspective on pediatric neurology, enabling diagnostic, therapeutic, and monitoring strategies tailored to the unique features of neurological illnesses in children. This review acknowledges the problems caused by delays in diagnosis, the limitations of conventional procedures, and the need for new, focused approaches, highlighting recent advances in nanoscale materials and smart nanocarriers. Specifically, this paper summarizes advances in nanomaterials that can overcome physiological barriers, such as the developing blood–brain barrier (BBB) and age-dependent pharmacokinetics. We discuss innovations in stimuli-responsive delivery systems, theranostic platforms, and multimodal nanohybrids designed for precise targeting and real-time treatment monitoring. Special emphasis is placed on pediatric-specific considerations, including developmental differences in immune and metabolic responses, the necessity for age-adjusted dosing, and the potential long-term safety implications of nanoparticle exposure. Transformative applications are explored in various pediatric neurological conditions, including brain tumors, epilepsy, neurodevelopmental disorders, and rare degenerative diseases, emphasizing both achievements and challenges in translation. This paper evaluates various regulatory, ethical, and societal factors, alongside the integration of converging technologies such as AI-driven nanoparticle optimization, brain organoids, and digital twins to accelerate personalized therapy development. Conclusively, this paper emphasizes the importance of interdisciplinary collaboration, pediatric-focused clinical trial designs, and sustained investment to fully realize the potential of nanotechnology in improving neurological outcomes for children.

## 1. Introduction

Neurological disorders in children represent a major global clinical challenge, characterized by limited therapeutic options, diagnostic delays, and age-dependent treatment responses [1,2,3]. Nanotechnology offers a transformative framework to address these impediments, bringing promise for precise diagnostics, targeted therapies, and real-time monitoring strategies tailored to the developing nervous system [4,5,6].

In pediatric oncology, nanotechnology has emerged as a promising instrument to enhance cancer treatment. It offers multiple benefits, such as precise medication delivery, decreased toxicity, and integrated immunotherapy [7]. These characteristics provide significant advantages in the management of particular pediatric neoplasms, including neuroblastoma, retinoblastoma, central nervous system (CNS) cancers, and musculoskeletal tumors [8]. Nanotechnology-based approaches, including customized nanocarriers and liposomes, have shown potential in targeted medication delivery with reduced toxicity for pediatric malignancies, such as acute lymphoblastic leukemia (ALL) and acute myeloid leukemia [9,10]. Nanovesicles, peptide-functionalized liposomes, and tumor vascular-targeting liposomes have been useful in the therapy of neuroblastoma. Moreover, nanomedicines, nanoparticle-based drug delivery systems, and nanotechnology-based miRNA treatments show potential for treating neuroblastoma [11,12].

Nanotechnology’s potential transcends cancer treatment, offering promise in the management of infectious diseases [13]. This facilitates tailored medication delivery for the treatment of malaria and leishmaniasis, minimizing toxicity while preserving efficacy. Nanoparticles have been employed in bioassays for the detection and management of schistosomiasis, and nanocarriers address antibiotic resistance and improve the efficacy of medications in infectious disorders [14,15]. Moreover, nano-biosensors have heightened sensitivity in identifying bacterial infections, facilitating novel strategies in addressing pediatric infectious illnesses. Additionally, nanotechnology has enhanced the detection and treatment of tuberculosis (TB) and human immunodeficiency virus (HIV) infections by improving targeted drug delivery, diagnostics, and treatment efficacy [16].

In pediatric medicine, nanotechnology provides novel ways to diagnose and treat diverse illnesses [17]. It has demonstrated potential in epilepsy, enhanced newborn screening, cardiovascular problems, neuroinflammation, neurodegenerative conditions, gestational diabetes, bone diseases, mosquito-borne illnesses, micronutrient deficiencies, vulvovaginitis, and additional areas. Overall, nanotechnology has shown potential in numerous preclinical and clinical pediatric applications, extending from targeted drug delivery and tissue engineering to personalized medicine and disease management [10]. In this context, personalized medicine refers to tailoring nanoparticle design, dosing, and treatment timing to a child’s individual physiological profile (i.e., body weight, organ maturity, and genetic variability in drug metabolism) rather than applying uniform, adult-derived protocols. This is especially relevant in pediatric neurology because the developing brain and BBB change rapidly with age, meaning a formulation optimized for a toddler may be unsuitable for an adolescent [18,19,20].

Given the unique challenges of the developing nervous system (e.g., dynamic maturation of the blood–brain barrier (BBB), age-dependent pharmacokinetics, and long-term safety concerns of nanoparticle exposure), focusing specifically on pediatric neurology is both timely and necessary. In this context, this review is based on a thorough investigation aimed at uncovering research at the crossroads of nanotechnology and pediatric neurology. This paper incorporates findings from preclinical and clinical studies, along with results from recent research articles that detail the use of nanotechnology for the diagnosis, monitoring, or treatment of neurological disorders in pediatric populations.

## 2. Pediatric-Specific Considerations in Nanoneurology

The BBB in children undergoes distinct developmental changes that influence the permeability and transport functions of the barrier. BBB is composed of various structural components, including tight junction proteins, endothelial cells, and pericytes. The establishment of these components and protective transport mechanisms occurs early in the developmental process [21,22]. This indicates that the BBB in newborns is effective at preventing substances from entering the brain. For instance, the blood vessels in the fetal and newborn brain are equipped with essential efflux transporters, yet expression levels and activity change as the brain matures. The variations in development indicate that the mechanisms by which nanomaterials enter the brain may change with age [23,24]. The CNS of an adult is generally capable of effectively eliminating nanoparticles. However, in a newborn, the process may be more straightforward, particularly if efflux transporter expression or function is diminished during that early stage of development [25,26].

The initial days following birth are particularly important. The placenta abruptly ceases its role in shielding the baby from medications and toxins present in the bloodstream, potentially increasing the baby’s brain’s susceptibility to these substances until the BBB is fully developed [27]. Furthermore, various stressors may have a greater impact on the integrity of the BBB during critical periods of brain development [25,28]. Researchers believe that heightened permeability observed during specific developmental stages may contribute to the onset of neurological disorders. Thus, understanding the age-dependent properties of the BBB is crucial, particularly from the perspective of nanoneurology.

Modifying the structure and function of the BBB may enhance the effectiveness of brain targeting in infants and children. For instance, aligning drug delivery with developmental stages or tailoring ligand targets to receptors that are prominently expressed in the immature barrier may prove beneficial [29]. As children grow, their immunological and metabolic systems undergo significant changes, which greatly influence the distribution, clearance, and efficacy of nanoparticles within the body. From an immunological perspective, young patients, particularly newborns and infants, possess immune systems that are still maturing [30]. Newborns possess a complement system that is not fully developed and have reduced levels of opsonizing antibodies. This may result in prolonged nanocarrier presence within the body in this age group [31,32], unforeseen immune responses, or accumulation [33], necessitating the use of “stealth” techniques such as PEGylation or biomimetic coatings [34]. Diverse functionalization methods for improving nanomaterials’ compatibility help regulate the immune system’s activity, preventing it from becoming overly active [35]. Additionally, they contribute to enhancing immune clearance by ensuring that nanoparticles remain biodegradable and non-harmful within the body over an extended period [34,35,36].

**Figure 1:**
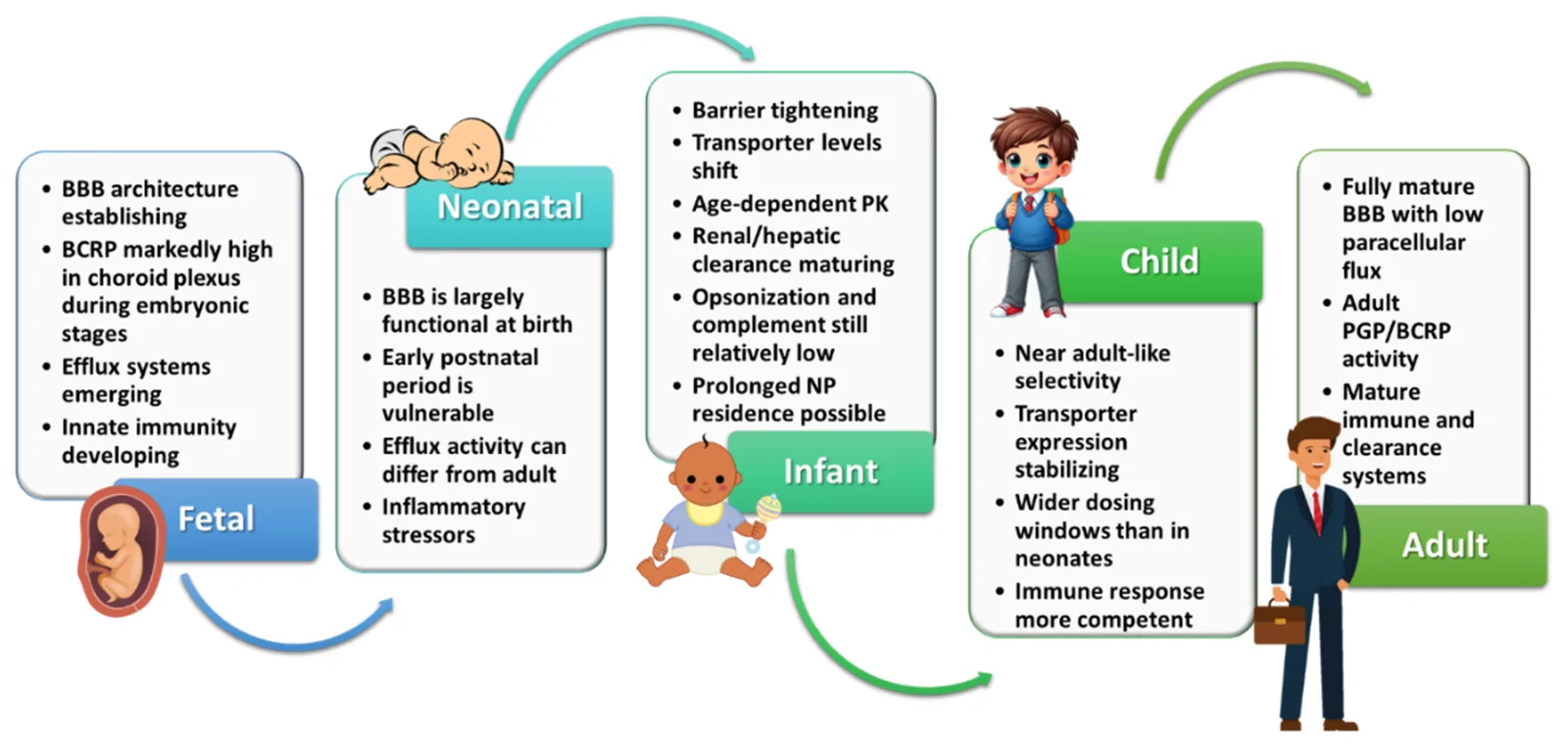
Developmental evolution of the pediatric BBB and immune system. Abbreviations: BBB, blood–brain barrier; PGP, P-glycoprotein; BCRP, breast cancer resistance protein; PK, pharmacokinetics. Created based on information from [23,24,25,26,27].

Figure 1 provides a visual overview of BBB and immune system evolution with age.

Pediatric nanoneurology is shaped by age-dependent features of the BBB, immune ontogeny, pharmacokinetics, metabolic enzyme maturation, renal clearance, dosing/PK modeling, and ethics. Table 1 presents key pediatric-specific factors in nanoneurology, highlighting their mechanism/application, relevance, and implications for nanocarriers.

When it comes to nanoneurology research, another aspect to consider is the use of animal models, which can offer essential insights into toxicity, pharmacokinetics, and therapeutic efficacy, thereby serving a vital function in drug development. Although this approach could be essential, numerous obstacles are associated with it [64,65]. Experimental animals must be in the same growth phase as the pediatric population under investigation; however, developmental timeframes vary among species. Moreover, the comparative rate of postnatal organ development differs among species, raising concerns due to the dynamic interplay of organ systems [65,66]. Temporal developmental disparities complicate the evaluation of possible toxicity in children through animal models. Preferably, the route of administration used in animal studies should align with the planned route in human investigations; however, the small size of juvenile animals presents technical difficulties in intravenous or cutaneous drug administration [65].

## 3. Innovations in Nanomaterials for Pediatric Neurological Applications

### 3.1. Pediatric Neural Interfaces

The human nervous system is a highly complex network comprising billions of neurons and glial cells connected through trillions of synapses. This intricate organization allows for long-distance electrical communication via axons and highly localized chemical signaling at synaptic junctions [8,67,68,69]. Understanding and interacting with such an intricate system is especially challenging in children, as their nervous systems are still developing and highly sensitive to external interventions.

To probe and modulate neural activity, researchers have developed a diverse range of neural interfaces capable of recording, stimulating, or regulating brain function both in vivo and in vitro [12]. Clinically, such technologies utilize arrays of several dozen electrodes that connect with the scalp or the brain’s surface to record spatiotemporal fluctuations in electrical potential, employing techniques referred to as electroencephalography (EEG) and electrocorticography (ECoG), respectively [8]. These devices can furnish critical information for the investigation and diagnosis of different neurological disorders, including Zika virus-induced microcephaly and epilepsy [70,71]. The resultant impulses may also serve as brain-machine interfaces for the control of prosthetics and other external devices. The electrodes serve as sources of electrical stimulation, capable of activating or deactivating specific brain networks to eradicate seizures and tremors [8,72].

In laboratory settings, human induced pluripotent stem cell (hiPSC)-derived neurons and three-dimensional organoids are increasingly used as models to study pediatric neurological diseases and drug responses [8,16,67]. However, conventional electrode systems (1D or 2D arrays) are limited in their ability to interact with such complex tissues [8]. To overcome these constraints, recent advances include flexible, mesh-like electronics and tissue-embedding nanomaterials, which provide more physiologically relevant three-dimensional interfacing with developing neural systems [71].

Table 2 presents an overview of significant innovations in nanomaterials specifically tailored for pediatric neural interfaces, emphasizing the development of advanced carrier designs, multifunctional platforms, and various material selections.

### 3.2. Smart Nanocarriers

**Figure 2:**
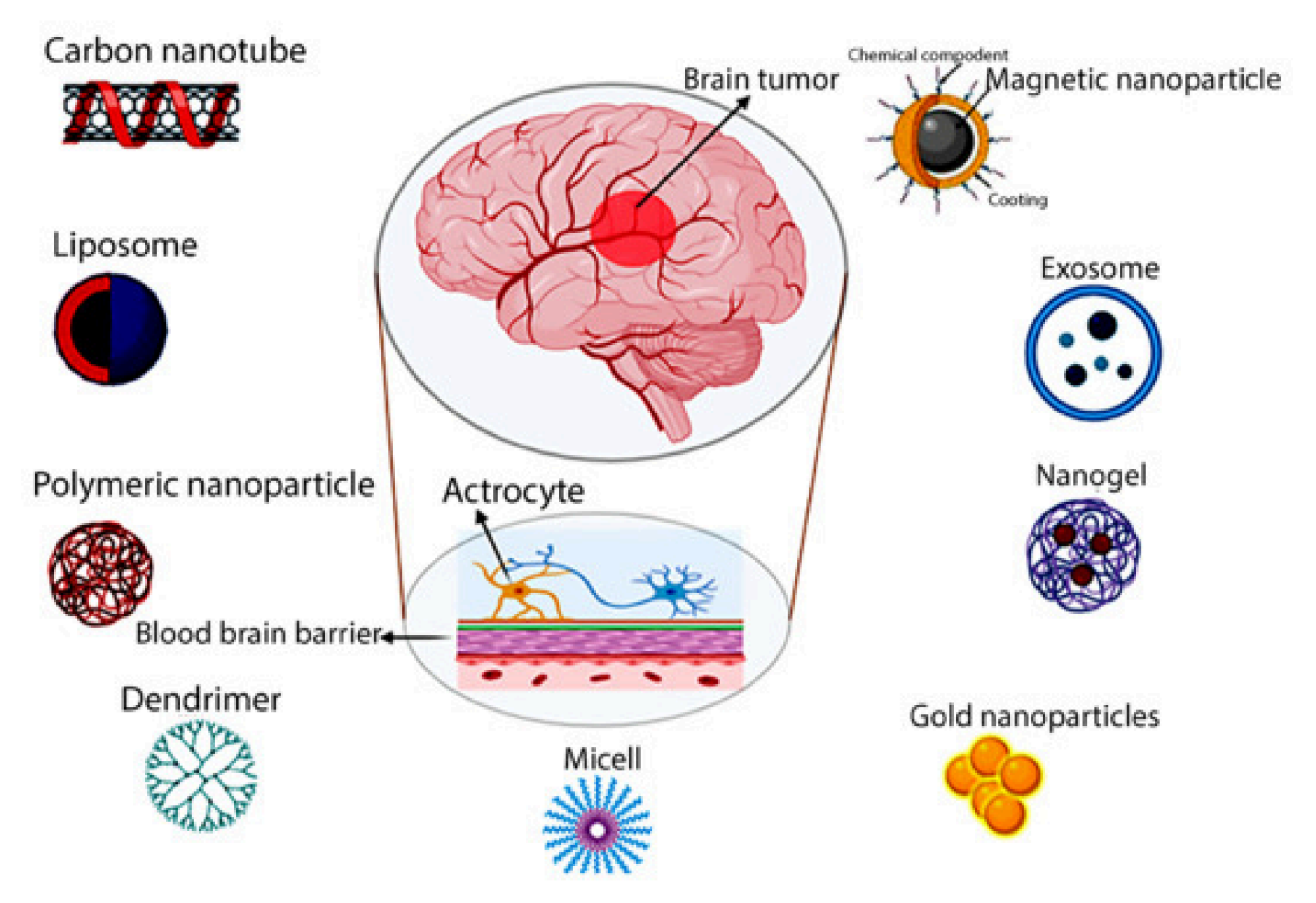
Innovative nanoparticles utilized in the therapy of brain cancer. Reprinted from an open-access source [27].

Smart nanocarriers enable context-aware delivery in the developing brain by coupling intrinsic and extrinsic triggers with pediatric-tailored materials. Essential approaches involve intelligent, responsive nanocarriers that deliver therapies exclusively to affected areas, multifunctional nanoparticles (presented in Figure 2) that integrate imaging and treatment simultaneously, and material selection (biodegradable versus long-circulating) tailored for growth and development [77].

**Figure 3:**
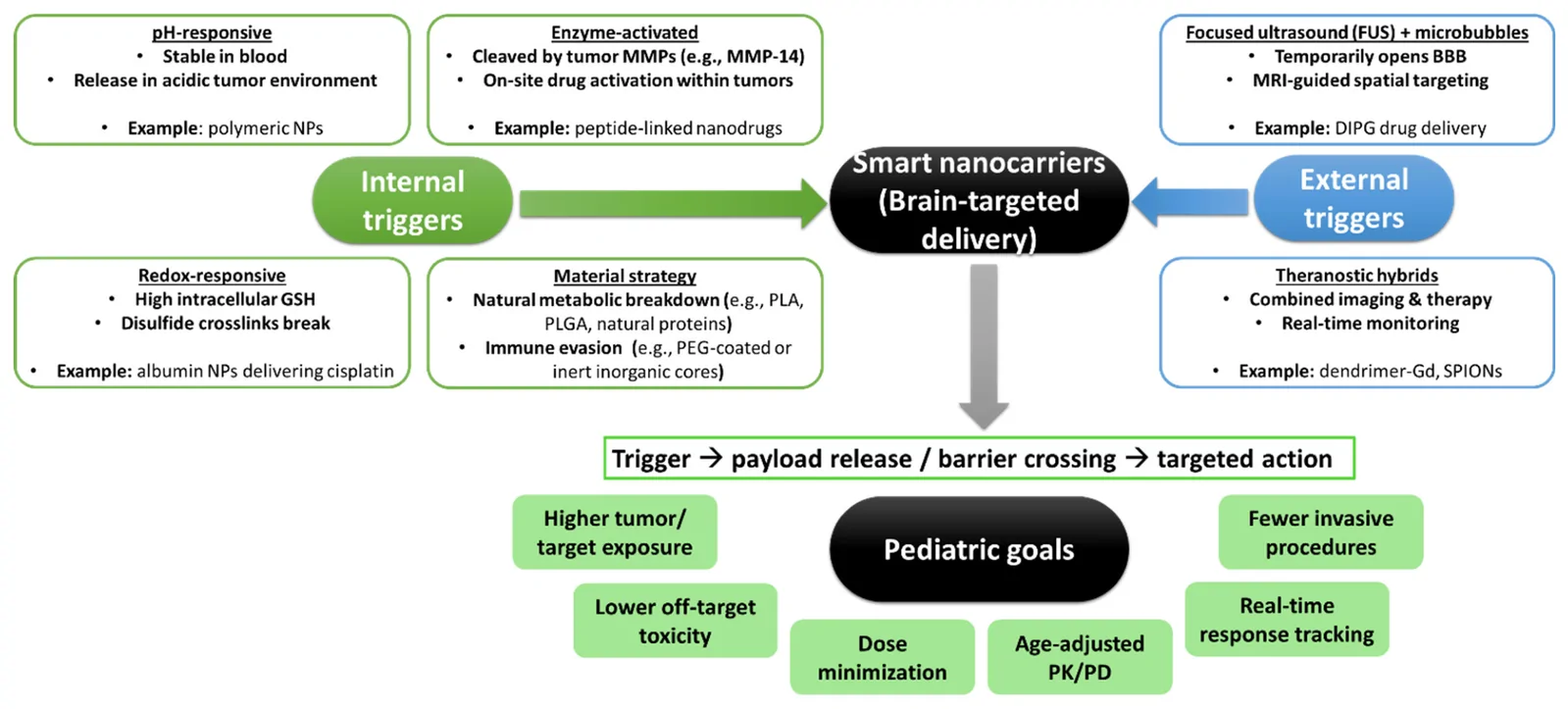
Smart nanocarriers in pediatric neural therapy. Abbreviations: BBB, blood–brain barrier; DIPG, diffuse intrinsic pontine glioma; FUS, focused ultrasound; Gd, gadolinium; GSH, glutathione; MMPs, matrix metallopeptidases; MRI, magnetic resonance imaging; NPs, nanoparticles; PEG, polyethylene glycol; PLA, polylactic acid; PLGA, poly(lactic-co-glycolic acid); PD, pharmacodynamics; PK, pharmacokinetics; SPIONs, superparamagnetic iron oxide nanoparticles. Created based on information from [80,81,82,83,84,85,86,87].

Advanced nanocarriers can be designed to respond to specific signals within the pediatric brain microenvironment, ensuring that drugs reach only the intended target [78]. As summarized in Figure 3, nanoparticles have been designed to remain stable in the circulation while allowing drugs to be released when they reach an acidic pH and/or higher levels of certain enzymes [67,79]. This response behavior successfully sends high amounts of medication to the right places, sparing healthy brain tissue that is still growing and lowering the risk of side toxicity [7,79].

External variables can also be used to help distribute drugs directly to the children’s brain [88]. One such example is focused ultrasound (FUS), a noninvasive technique that uses ultrasonic waves to temporarily create holes in the BBB. This allows nanoparticles or medications to be delivered to specific parts of the brain with great accuracy. Studies on diffuse intrinsic pontine glioma (DIPG), a devastating brain tumor in children, have shown that magnetic resonance imaging (MRI)-guided focused ultrasound makes it much easier for drugs to reach the tumor. This approach improved drug delivery to the CNS and was well-received, suggesting that concentrated ultrasound-induced BBB breakdown is both safe and effective for children [50,89].

**Figure 4:**
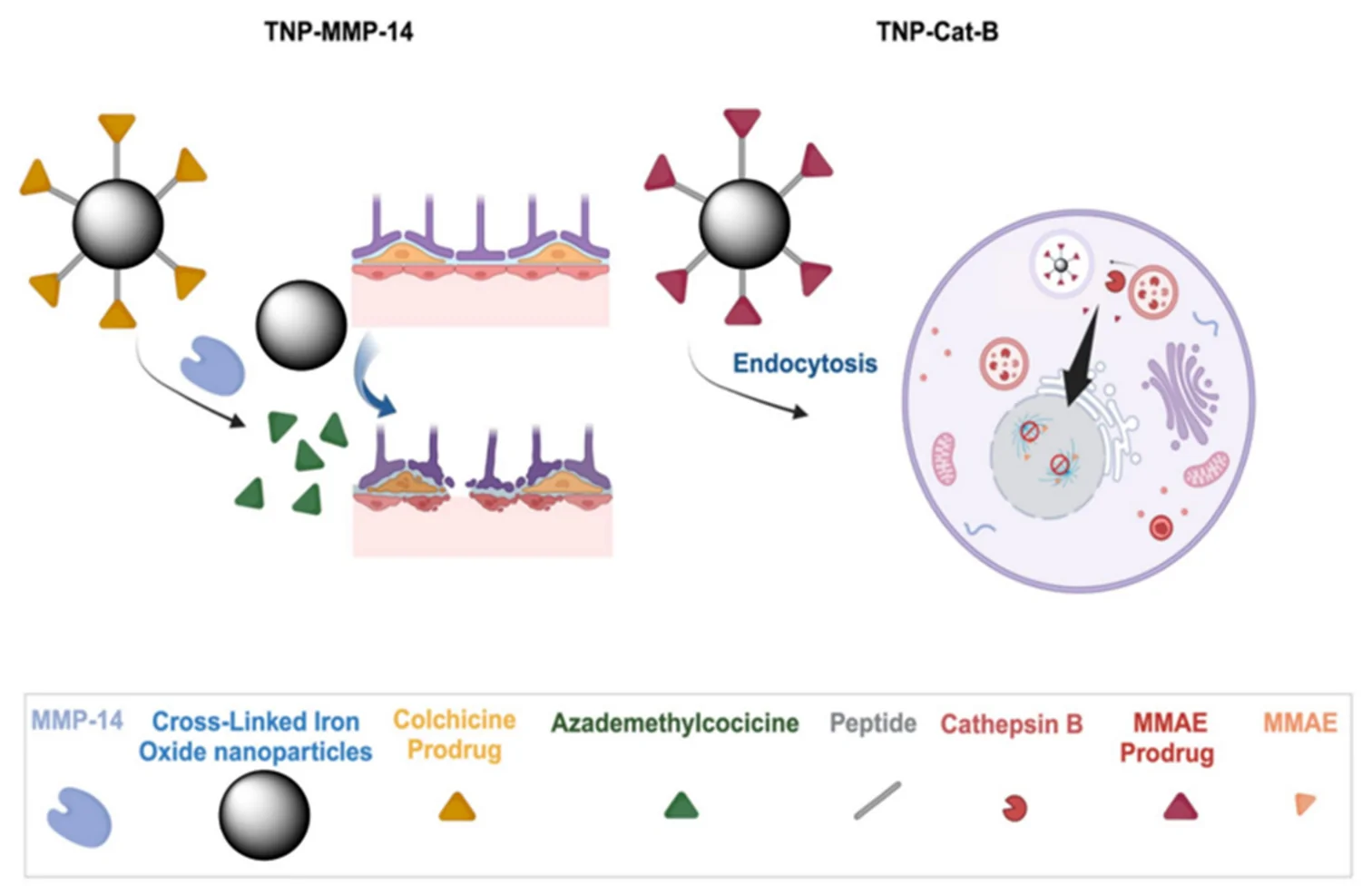
Dual-enzyme activatable theranostic nanoparticles (TNPs) for image-guided glioblastoma therapy. Reprinted from an open-access source [99].

Theranostic nanoparticles combine diagnostic imaging and therapeutic capabilities within a single platform. This combined ability is especially significant in pediatric care, where the goal is to monitor treatment in real time and reduce the need for invasive procedures [90,91,92]. These modern approaches allow real-time monitoring of how well a child’s brain tumor is responding to treatment and how well the drugs are getting to the tumor right away [93,94,95,96]. Thus, theranostic nanoparticles provide a “see and treat” approach that makes things safer and more successful. For example, using MRI to guide drug release enables the visualization of accurate nanocarrier delivery, which is very important, especially in the context of the growing brain [78,94,97,98]. This approach can also be observed in Figure 4.

A range of theranostic nanohybrids of interest for pediatric research has been reported in the literature [94]. For instance, the modification of dendrimer-based nanocarriers has involved integrating gadolinium (Gd) chelates with fluorescent dyes. This integration allows for high-resolution visualization of brain tumor boundaries using both MRI and optical fluorescence techniques, while also delivering medication [91,100]. Changes in MRI signals can measure the degree to which drugs are delivered to brain tumors, allowing a pediatric neuro-oncologist to monitor nanodrug delivery to a child’s tumor in real time and facilitate necessary dosage adjustments [72,101].

Additionally, theranostic nanoprobes have been developed that release their drugs only inside tumors when enzymes are present, while being tracked using MRI technology. Two separate probes were used in a preclinical model of brain tumors to monitor treatment progress in real time using MRI [90,101]. One nanoparticle messed with the tumor’s blood vessels, making it easier to get more drugs to the tumor. The other nanoparticle was made to precisely kill cancer cells. The MRI showed that both probes were active only within the tumor. These examples show how multimodal nanohybrids can make monitoring brain therapy in kids more accurate and effective, highlighting the promise of giving each child safe, minimally invasive therapies tailored to their needs [93].

Interesting prospects in pediatric oncology also arise from liposomal drug formulations that extend the half-life and retention time of nanoparticles, significantly enhancing drug delivery to tumors and leading to better therapeutic outcomes [102].On the other hand, extended persistence raises concerns about tissue accumulation and the possibility of long-term damage [50,103]. The mononuclear phagocyte system in children is not yet fully mature, which hinders its capacity to eliminate certain nanoparticles compared with that of adult patients. There are valid concerns regarding the potential effects on growth and organ function that remain unclear when non-biodegradable nanoparticles, such as gold or silica, accumulate in organs like the liver, spleen, or lungs after repeated administrations [50,51]. Moreover, the ongoing application of non-biodegradable polymeric nanoparticles has been linked to localized immune reactions or quicker elimination during subsequent administrations, a phenomenon known as the “ABC phenomenon” in PEGylated therapies [52,53]. A nanoparticle coated with PEG, which gradually deteriorates, is often used to balance the two. As a result, the nanoparticle will eventually leave the child’s body after fulfilling its role in delivering medicine [93].

### 3.3. Blood–Brain Barrier-Targeted Delivery Systems

Apart from the intrinsic- and extrinsic-trigger mechanisms mentioned above, receptor-mediated targeting provides a complementary way to bridge the pediatric BBB by leveraging endogenous transport systems rather than physical disruption or passive diffusion. Transferrin-targeted nanoparticles use the high expression of transferrin receptors on brain capillary endothelial cells to promote receptor-mediated transcytosis. Nanoparticle formulations functionalized with anti-transferrin receptor antibodies or ligands have been shown to significantly enhance brain uptake compared to non-targeted formulations. Switching from bivalent to monovalent antibody binding can lead to several-fold increases in brain uptake. Low affinity binding appears to favor dissociation from the receptor and subsequent passage into the brain parenchyma rather than retention at the capillary wall [104,105].

Glutathione-targeted nanoparticles are a second, well-characterized, receptor-mediated pathway. Glutathione-PEGylated liposomes (G-Technology^®^) use the glutathione transporters expressed at the BBB. In rat studies, glutathione-coated liposomes led to about 4-fold higher brain concentrations of a fluorescent tracer compared to non-targeted PEGylated liposomes, and also 1.8-fold higher cellular uptake by brain endothelial cells in vitro [104]. Receptor-targeted strategies are more selective and offer potentially lower-dose delivery than non-targeted nanoparticles that enter the CNS by passive diffusion or transient physical disruption of the BBB (e.g., the focused-ultrasound approach described above), which is an attractive feature particularly in pediatric populations where the aim is to minimize systemic and off-target exposure. However, receptor expression at the BBB may vary with age and disease state, and pediatric-specific efficacy and safety data for both transferrin- and glutathione-targeted platforms are still limited, representing a critical avenue for future research before translation of these strategies to pediatric neurological indications.

Translating transferrin-targeted platforms into robust clinical formulations also faces practical constraints. The BBB is also continuously saturated by circulating endogenous transferrin, which competes with transferrin-conjugated nanoparticles for binding. This competition is one reason several research groups have chosen anti-transferrin receptor antibodies instead of transferrin itself as the targeting ligand [106]. It is also important to achieve the right nanoparticle avidity for the transferrin receptor: nanoparticles coated with too much transferrin are inclined to stick to the luminal surface of brain endothelial cells instead of being released into the brain parenchyma, while suitably tuned, lower avidity formulations are more likely to detach from the receptor and complete transcytosis. More generally, receptor saturation and inefficient intracellular trafficking have been identified as ongoing practical limitations of transferrin receptor-targeted platforms, underscoring that translation of these systems into reproducible pediatric formulations will require careful optimization of ligand density and binding affinity, not receptor targeting alone [107,108].

## 4. Transformative Applications: From Bench to Bedside

**Figure 5:**
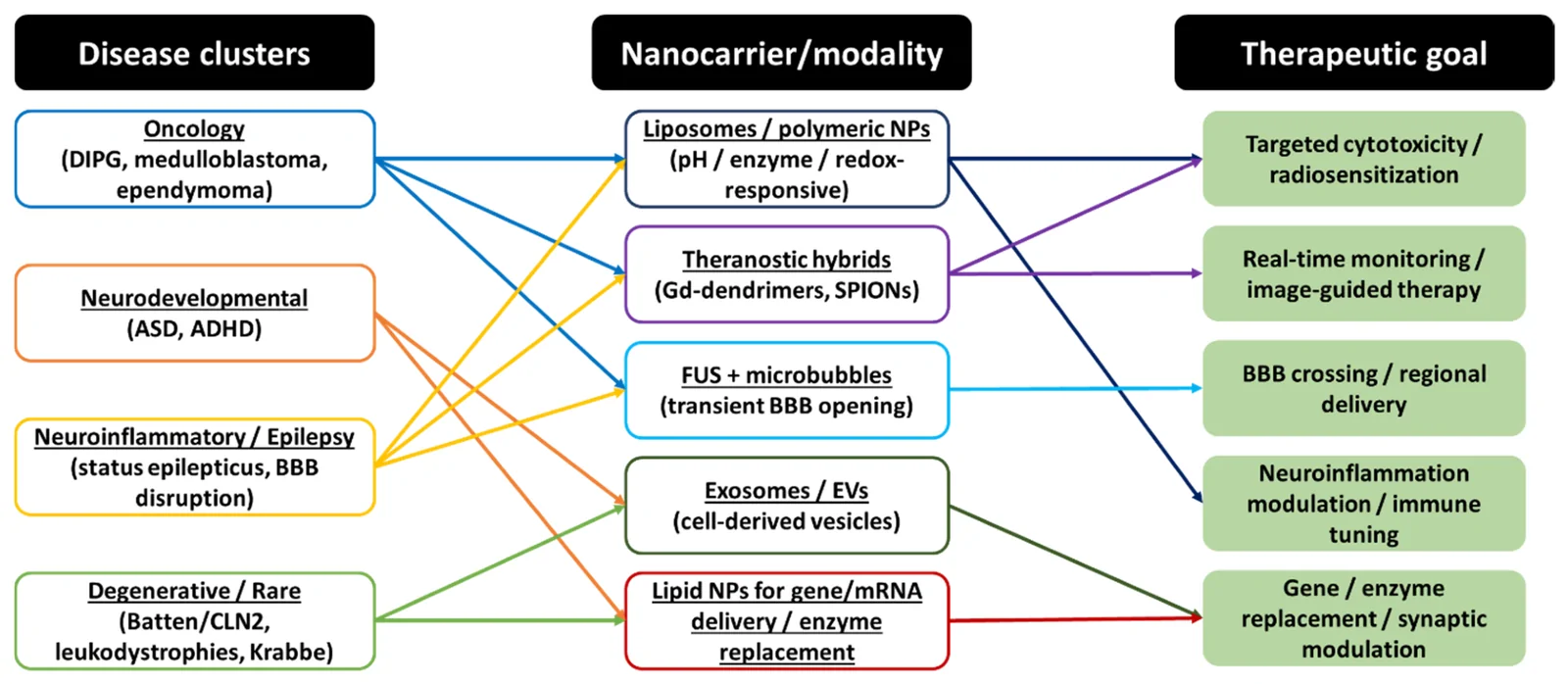
Mechanistic map of nanoparticle interventions across pediatric neurological diseases. Abbreviations: ADHD, attention-deficit/hyperactivity disorder; ASD, autism spectrum disorder; BBB, blood–brain barrier; DIPG, diffuse intrinsic pontine glioma; EVs, extracellular vesicles; FUS, focused ultrasound; Gd, gadolinium; LNP, lipid nanoparticle; SPIONs, superparamagnetic iron oxide nanoparticles.

Translation in pediatric nanoneurology hinges on matching the right delivery strategy to the right pathophysiology—tumor selectivity in oncology, synaptic/immune modulation in neurodevelopmental disease, and barrier control in epilepsy and rare disorders. In this respect, Figure 5 offers a visual summary for this section, mapping disease clusters to nanocarrier modalities and their primary therapeutic goals.

### 4.1. Pediatric Brain Tumors

As illustrated in Figure 5, evidence converges on three complementary strategies concerning pediatric brain tumors: (i) internal triggers in liposomal/polymeric systems for tumor-selective release, (ii) external guidance via MRI-navigated FUS to transiently open the BBB, and (iii) theranostic hybrids that couple imaging to delivery for real-time adaptation.

Several of them have advanced to the stage of clinical trials. For example, liposomal chemotherapy formulations have been evaluated in pediatric populations: liposomal irinotecan (topoisomerase inhibitor) has been tested in Phase 1 for neuroblastoma and pediatric brain tumors (NCT00019630), and several clinical and preclinical studies have evaluated liposomal doxorubicin in children [109,110,111]. Even though liposomes are engineered to reduce systemic toxicity and promote intratumoral deposition of drugs, a completed Phase 1 of liposomal doxorubicin in children with brain tumors revealed only a modest therapeutic impact. Moreover, a different pediatric trial involving a similar formulation was halted early, highlighting practical and biological hurdles. Likewise, a trial involving the iron oxide nanoparticle MRI agent Combidex was terminated before completion once the product was no longer available, illustrating supply-chain constraints that can derail otherwise informative investigations. These experiences also emphasize that some nanoparticles that produced desirable effects in murine models frequently failed to show sufficient tumor penetration or clinical advantages in human trials, emphasizing the complexity of clinical implementation [110].

A groundbreaking approach targets DIPG through a convection-enhanced nanoparticle suspension of panobinostat (MTX110), which enables direct infusion into the brainstem tumor. Specifically, a recent Phase 1 trial (NCT04341311) has been completed, demonstrating that nanocarriers can be safely administered via intratumoral catheters. A subsequent trial (NCT03566199) is in the process of recruiting patients to evaluate MTX110 efficacy with co-infused gadolinium for real-time distribution imaging, with early data suggesting higher local drug concentrations than systemic therapy [112].

Additional trials involve the use of gold nanoparticles as radiosensitizers in gliomas, enhancing the DNA damage inflicted on tumor cells during radiation treatment [113]. Preclinical studies conducted on mice demonstrated that the combination of PEG-coated gold nanoparticles and radiotherapy notably improved survival rates in orthotopic glioma models [114]. Nonetheless, applying this to pediatric patients necessitates a thorough assessment of toxicity and long-term neurodevelopmental surveillance. Overall, targeted nanotherapies for pediatric brain tumors have demonstrated promising proof-of-concept successes but remain constrained by setbacks related to safety concerns, manufacturing challenges, and insufficient efficacy, reinforcing the need for better targeting strategies and predictive biomarkers to rapidly identify responders [115,116].

Examples of these clinical experiences have been summarized for better clarity in Table 3 at the end of Section 4.6. Overview discussion.

### 4.2. Neurodevelopmental Disorders

Beyond oncology, nanotechnology is explored for neurodevelopmental disorders, including autism spectrum disorder (ASD) and attention-deficit/hyperactivity disorder (ADHD) [114]. These conditions involve dysregulated neurotransmission and neuroimmune signaling that are difficult to modulate with conventional pharmacotherapy alone. Nanoscale delivery systems offer targeted control of neurotransmitters and inflammation with higher anatomical precision [117]. In ADHD, where cortico-striatal dopamine and norepinephrine signaling is impaired, intranasal administration of atomoxetine via solid lipid nanoparticles (SLNs) improved drug delivery to the brain, resulting in more than 50% direct transport of the drug to the brain, in contrast to nearly 0% for oral dosing. This led to increased brain concentrations of atomoxetine, suggesting potential dose-sparing and fewer systemic effects associated with nose-to-brain nanoparticle systems [118,119]. More speculative approaches include nanoporous minerals (e.g., clinoptilolite nano-zeolites) for detoxifying the brain environment in individuals with ADHD, potentially enhancing cognitive performance if lead exposure was a contributing factor, or as carriers of dopamine, enabling the controlled release of this neurotransmitter to specific brain regions [116,120].

On a different note, children with ASD may exhibit persistent brain inflammation and immune dysregulation [121,122,123]. Preclinical research proposed using human stem cell-derived exosomes as a therapeutic strategy to mitigate neuroinflammation [124,125]. Perets et al. [126] examined the intranasal delivery of exosomes (~100 nm diameter) enriched with anti-inflammatory and growth factors to BTBR mice, an accepted model for evaluating autism-like behaviors exhibiting all core symptoms of ASD [112]. The nanocarriers demonstrated brain entry, notable behavioral improvements, and decreased neuroinflammation [126]. These findings support the concept of “cell-free” nano-biologics that leverage the body’s inherent signaling mechanisms to reestablish neural balance and immune homeostasis [127].

Overall, nanoplatforms designed for ASD and ADHD are at an early stage of development, but show significant potential for overcoming BBB constraints, enabling synaptic-level modulation of neural biochemistry and addressing peripheral factors (e.g., immune signals, toxins), which are often beyond the reach of conventional treatments [118,128,129,130]. Relevant examples from studies related to the application of nanotechnology in neurodevelopmental disorders have been synthesized in Table 3 at the end of Section 4.6. Overview discussion.

### 4.3. Epilepsy/Neuroinflammatory Phenotypes

In children, epilepsy reflects a bidirectional cycle of irregular electrical activity and neuroinflammation. Nanomedicine addresses both aspects of this issue by enhancing CNS delivery of antiseizure medications and mitigating inflammatory processes that may worsen seizures [127,131]. A significant advancement is the application of nanocarriers to enhance drugs’ ability to traverse the BBB, as many antiseizure drugs are incompletely brain-penetrant or are expelled by efflux transporters, particularly during prolonged seizures when BBB permeability fluctuates. Encapsulation can protect these drugs from being expelled and facilitate their transport into the brain tissue [132,133]. For example, 125 nm polymeric nanoparticles loaded with lamotrigine elevated drug concentrations in the brains of rats compared with oral free drug administration and ensured enhanced traversal and retention across the BBB. Similarly, nano-encapsulated phenytoin revealed improved efficacy when tested on a rat model of epilepsy, as the animals exhibited reduced seizure scores and severity [77]. Broad surveys also report nanoformulations with either enhanced anticonvulsant potency or diminished toxicity, including gold nanoparticle-conjugated lacosamide (improvements in seizure-related EEG readings) and dendrimer-based carriers for carbamazepine (enhanced drug solubility while minimizing side effects) [134,135]. By overcoming BBB and multidrug resistance constraints, nanocarriers present a compelling strategy for children with refractory epilepsy [135].

Another area of exploration for nano-interventions involves targeting the neuroinflammatory loop: seizures activate microglia and pro-inflammatory cytokine cascades that further disrupt the BBB and promote additional seizures [136]. Nanotechnology offers innovative tools that can disrupt this cycle. Biodegradable nanoparticles can transport anti-inflammatory medications, such as steroids, NSAIDs, or experimental inhibitors, directly to immune cells in the brain exhibiting heightened activity [137]. For instance, liposomal curcumin mitigated glial activation and significantly lowered IL-1β, IL-6, and other cytokine levels in status models versus free drug [129]. Moreover, some nanomaterials possess inherent anti-inflammatory and modulatory properties: gold nanoparticles (can shift microglia from a pro-inflammatory state to a neuroprotective one [24]), carbon nanotubes (may interact with neuronal membranes, influencing electrical activity or facilitating neuronal repair processes [138]). Other related concepts include magnetothermal stimulation, which employs magnetic nanoparticles to soothe neurons in a seizure focus and support regeneration in brain injuries linked to epilepsy [109].

Moreover, nanomedicine aids in the surveillance of BBB disturbances associated with epilepsy. Iron oxide nanoparticles (e.g., ferumoxytol), as MRI tracers, have mapped compromised regions of the BBB in epileptic foci [139]. Conversely, nanoparticles may also play a protective role for the BBB; by scavenging reactive oxygen species (ROS) or delivering agents that stabilize the barrier, they could mitigate the breakdown that often follows prolonged seizures. For example, antioxidant-loaded polymer nanoparticles have demonstrated neuroprotective properties in models of traumatic brain injury, with clear relevance to status epilepticus and post-ictal barrier repair [132].

A series of discussed examples has been summarized in Table 3 at the end of Section 4.6. Overview discussion.

### 4.4. Rare Degenerative Disorders

Rare pediatric neurodegenerative disorders, including leukodystrophies (e.g., Krabbe’s and adrenoleukodystrophy), Batten disease (neuronal ceroid lipofuscinosis), and related metabolic encephalopathies, pose significant treatment challenges, arising from single-gene defects causing enzyme loss or toxic protein accumulation [140].

For Batten disease (CLN2), the approved therapy requires intraventricular enzyme infusion due to poor BBB permeability. Researchers explore nanoparticles or extracellular vesicles (EVs) to deliver enzymes or gene payloads less invasively [136]. According to a 2025 systematic study, nanoparticles and natural EVs can pass the blood-brain and blood-ocular barriers, supporting prospects for intravenous strategies in Batten disease [127]. In cellular and animal models, liposomes and polymer nanoparticles have effectively transported TPP1 enzyme to neural cells, reducing storage material in CLN2. Similarly, in Krabbe disease, research currently centers on using lipid nanoparticles for the targeted delivery of mRNA encoding the enzyme to prevent myelin degradation in oligodendrocytes in the brain [138]. Moore et al. [141] highlight lessons from Krabbe for CNS nanodelivery, including the successful transport of gene therapy vectors and enzyme-loaded nanoparticles across the BBB in preclinical models. Recent broader perspectives also suggest that nanotechnology could bridge therapeutics and affected regions in rare pediatric disorders [142]. Yet real-world translation remains nontrivial: complex nanoparticle architectures interface with a developing organism optimized to exclude atypical particulates, and pediatric generalization from animal success is uncertain [129].

To date, no nanoparticle therapy for pediatric neurodegenerative diseases has successfully passed clinical trials, with challenges related to dosage, targeting specificity, and long-term safety persisting as significant obstacles [112,118,143]. A 2021 virus-loaded nanoparticle trial for a rare encephalopathy faced a pause due to inflammation concerns, illustrating the risk of triggering the body’s immune responses. Furthermore, manufacturing scale, purity, and consistency requirements pose additional hurdles often underappreciated in early reports [139,144,145,146].

Nonetheless, selective milestones signal feasibility. For instance, in 2018, a unique nanoparticle-based antisense oligonucleotide, known as Milasen, was developed specifically for one patient suffering from Batten disease, effectively stabilizing her condition [129]. Furthermore, enzyme-loaded nanoparticles have been shown to prolong survival and enhance neurological function in mice models of Sanfilippo syndrome [147,148].

All in all, the field stands at a pivotal juncture between experimental promise and clinical pragmatism. Skeptics rightly warn that certain initial assertions were overly optimistic, as addressing a child’s extensive brain degeneration will not be as straightforward as administering a nanoparticle injection [134]. Nevertheless, mechanism-guided advances continue, opening previously “undruggable” CNS targets to therapeutic exploration [109].

### 4.5. Other Applications

Besides therapeutic approaches, nanotechnology has enabled the development of highly sensitive diagnostic techniques for pediatric neurological disorders that outperform conventional imaging and cerebrospinal fluid sampling [149]. “Brain liquid biopsy” approaches involve identifying disease markers in blood or urine through nanoscale technologies [150]. For instance, tumor-derived exosomes (30 and 150 nm vesicles) can be extracted from blood samples and examined for RNA/protein signatures. In pediatric gliomas, exosomal microRNAs have demonstrated significant diagnostic accuracy (e.g., AUC of approximately 0.81 in differentiating high-grade gliomas) [149,151]. Researchers have created nano-enhanced sensors to improve analytical sensitivity. Huilin Shao et al. [149] introduced a “EZ-READ” platform that captures EV RNAs from blood, detecting approximately 9 RNA copies and achieving around 90% accuracy in identifying glioblastoma. Similarly, Yang et al. [152] developed a multiplex optical biochip using magnetic nanochains to enhance detection signals and allow multi-marker exosome detection in just 30 min. Collectively, these nano-biosensors lower the limit of detection for faint disease signals, enabling clinicians to identify pediatric neuro-oncological diseases at a much earlier stage than traditional assays.

Nanotechnology is also transforming neuro-imaging, enabling earlier and more accurate diagnoses [153,154]. Nanoscale contrast agents capable of traversing the BBB can reveal subtle lesions that traditional imaging techniques often overlook. In a “brain tumor painting” technique, 33 nm chlorotoxin-targeted, infrared-labeled nanoparticles crossed the BBB and illuminated brain tumors on MRI/optical scans with crisper margins than conventional Gd, reportedly approaching tenfold resolution gains and enabling detection of sub-millimeter foci [109,148]. Similar nanoprobe techniques are being developed to enhance signals from proteins associated with rare neurodegenerative diseases and to identify minor inflammatory changes occurring in the brain, with encouraging preclinical results [118,155].

### 4.6. Overview Discussion

**Figure 6:**
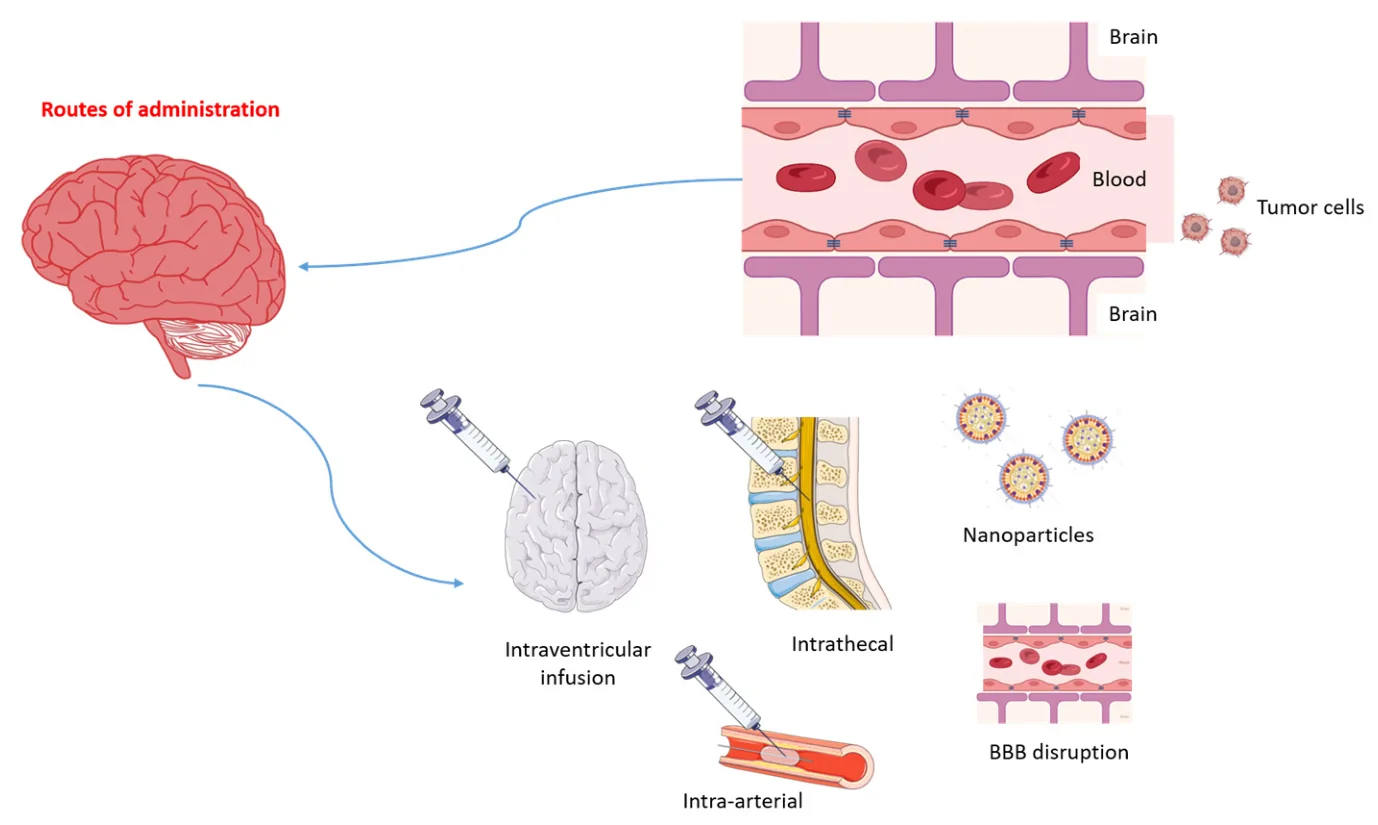
Illustrative examples of various pathways for delivering substances to the central nervous system. Created based on information from [109,156].

Overviewing the above-mentioned findings, Table 3 consolidates representative clinical and preclinical nanotherapeutic studies across pediatric neurological indications, standardizing the essential variables: condition, nanotechnology type, route, key findings, and developmental stage, offering an at-a-glance perspective over the applications of nanotherapeutics in pediatric neurology. Complementing this summary, Figure 6 illustrates several routes of administration to the CNS, evidencing common pathways for nanoparticle-mediated drug delivery to the brain.

Read together, the table and figure foreground the translational patterns that matter most in children: (i) barrier-modulation strategies (CED, FUS, intranasal) that increase regional exposure; and (ii) formulation-driven gains (liposomes, polymeric NPs, EVs, LNPs, AuNPs) that alter pharmacokinetics and toxicity.

**Table 3:** Summary of Clinical and Preclinical Trials in Pediatric Neurology.

Condition	Nanotechnology Type	Route	Key Finding(s)	Stage	Refs.
DIPG (brainstem glioma)	Convection-enhanced nanoparticle panobinostat (MTX110)	Intratumoral CED (with MRI co-infusion)	Feasible local delivery; higher intratumoral drug levels vs. systemic; ongoing efficacy evaluation	Phase I/ongoing follow-up	[112]
Pediatric gliomas	FUS + microbubbles with systemic agents	IV + MRI-guided FUS	Transient BBB opening enabling regional drug uptake; acceptable acute safety profile	Early clinical/feasibility	[50,85,89]
Pediatric glioma (imaging)	Iron-oxide (ferumoxytol) contrast	IV	Enhanced lesion visualization/BBB disruption mapping; pediatric study halted due to product supply	Clinical/terminated	[139]
Pediatric brain tumors	Liposomal doxorubicin	IV	Mixed results; one Phase I modest benefit and another halted; illustrates translation challenges	Phase I/halted	[109]
Pediatric neuroblastoma/brain tumors	Liposomal irinotecan	IV	Evaluated in Phase I pediatric settings	Phase I	[109]
Glioblastoma (radiosensitization concept)	PEG-coated gold nanoparticles	IV + radiotherapy	Improved survival in orthotopic mice; pediatric safety requires careful toxicology	Preclinical	[113,114]
ADHD	Atomoxetine solid-lipid nanoparticles	Intranasal	>50% direct brain transport via olfactory routes vs. near 0% oral; higher brain levels, potential dose sparing	Preclinical (PK in rats)	[118]
ASD (mouse model)	Human stem-cell exosomes (~100 nm)	Intranasal	Reduced neuroinflammation; improved autistic-like behaviors in BTBR model	Preclinical	[126,127]
Epilepsy	Lamotrigine polymeric NPs (≈125 nm)	Systemic	Higher brain exposure and longer residence vs. free drug	Preclinical	[77]
Epilepsy	Phenytoin nano-encapsulation	Systemic	Lower seizure scores vs. same dose free drug; improved brain uptake	Preclinical	[77]
Epilepsy (multiple agents)	Dendrimer/carrier conjugates; AuNP-lacosamide	Systemic	Enhanced anticonvulsant potency or reduced toxicity; EEG improvements in rats	Preclinical	[134,135]
Rare degenerative (Batten/CLN2)	Enzyme/gene delivery via liposomes/polymeric NPs/EVs	Systemic/CNS routes	Brain delivery of enzymes/genes across barriers in models; improved survival/neurological function in some studies	Preclinical/translational outlook	[127,136,138,140,141,142,144,145,146]

Abbreviations: CED, convection-enhanced delivery; FUS, focused ultrasound; EVs, extracellular vesicles; IV, intravenous; PK, pharmacokinetics.

As summarized in Table 4, more than half of the nanotherapeutic platforms discussed in this review remain at the preclinical stage. Among the minority that reached clinical trials, three encountered early termination or only modest efficacy. This distribution underscores that, while the field shows broad conceptual promise, only a small subset of approaches are close to clinical readiness—a gap this review aims to make explicit rather than obscure.

Beyond therapeutic efficacy, translation from bench to bedside requires robust characterization of nanoparticle safety. Long-term toxicity is poorly established in pediatric populations: nanoparticles tend to concentrate in the liver, spleen, and kidneys, and repeated treatment with PEGylated or protein-coated nanoparticles can elicit anti-drug antibody responses or faster blood clearance. The pediatric immune system is still developing and may respond differently to these formulations than the adult immune system. There is less pediatric-specific biodistribution and immunogenicity evidence, which is a significant gap for regulatory confidence [157].

## 5. Regulatory, Ethical, and Societal Implications

Presently, pediatric regulatory frameworks in both the United States and Europe advocate for trials that prioritize children, yet they were not designed specifically for nanomedicines. In the US, the Best Pharmaceuticals for Children Act (BPCA) and the Pediatric Research Equity Act (PREA) encourage or mandate studies on pediatric populations. Similarly, in the EU, Pediatric Regulation (EC No. 1901/2006) requires a Pediatric Investigation Plan (PIP), unless a waiver is provided. These regulations have enhanced the inclusion of children but do not specifically address the unique considerations of nanotherapeutics, such as extended tissue retention or new toxicity profiles [58].

Age stratification is a fundamental element of trial design as categorized by ICH E11 and EMA guidance; several groups are distinguished: neonates (0–27 days), infants (1–23 months), children (2–11 years), and adolescents (12–18 years). Organizing enrollment according to these age groups helps ensure that dosing, endpoints, and safety windows are developmentally appropriate [58,59,152,158]. Nonetheless, the youngest cohorts are often excluded due to safety concerns, leaving critical evidence gaps in neonates and young infants; adaptive designs that sequence enrollment from older to younger groups after predefined safety gates can mitigate this gap and distribute benefits more equitably over time [60,62]. One of the major challenges in pediatric nanomedicine is dose translation, which involves how to translate doses established in adult or animal models to children. Nanoparticle pharmacokinetics are affected by additional age-dependent factors—reticuloendothelial system maturity, plasma protein binding and blood–brain barrier permeability—that vary non-linearly during development, unlike small molecule drugs, for which allometric scaling based on weight or body surface area is relatively well characterized. Therefore, a simple weight-based extrapolation may cause over- or underdosing in several pediatric populations, especially neonates and infants. Physiologically based pharmacokinetic (PBPK) modeling is a validated approach to improve pediatric dose translation for traditional drugs. Formulation-specific PBPK models incorporating nanoparticle-specific parameters (e.g., particle size distribution, dissolution behavior) are beginning to be developed. Still, nanoparticle PBPK models that are specifically tailored to pediatrics are rare and represent a critical research need [159].

Creating suitable endpoints for pediatric trials presents an additional challenge. Traditional adult efficacy endpoints (e.g., tumor size reduction, changes in neurologic scores) are frequently inadequate in children, where long-term developmental outcomes matter. Trials should consider pediatric-relevant endpoints such as cognitive development, academic performance, or quality of life at later stages. Despite agency recognition of these needs, there remains a disparity in the standardization of these endpoints across various trials [58,59]. One possibility is to integrate surrogate markers (e.g., neurodevelopmental biomarkers, MRI readouts of regional delivery) to evaluate the effects of nanodrugs more promptly, thereby minimizing the dependence on extended follow-up periods [160].

Ethically, pediatric nanotherapeutics are found at the intersection of uncertainty and vulnerability. Consent models must reconcile legal realities (i.e., children cannot provide full consent) with respect to the child’s developing autonomy. The best compromise is parental permission plus child assent when they are of an appropriate age, with attention to the risk of undue influence on families under stress [54,55,57,58,161,162]. Enhancements to consent protocols include creating materials tailored to different age groups (e.g., plain language, child-friendly illustrations) [56], decision aids designed for parents, and engaging independent pediatric advocates or ethics consultants on high-risk trials to insulate decisions from distress and optimism bias [57]. From an ethical standpoint, it is important to respect a child’s dissent when the circumstances do not pose a threat to their life. According to one guideline, a minor’s refusal may only be set aside if the trial presents a potential lifesaving advantage and there are no viable alternatives available [58].

Furthermore, regulatory frameworks such as 45 CFR 46 (Subpart D in the US) [163] define the permissible risk levels for pediatric research. Specifically, if a trial does not provide any direct benefit, it is required to present no more than minimal risk or a slight increase above minimal risk to receive ethical approval. Nanotherapeutics that carry significant risks yet show promise should typically be assessed within the framework of serious diseases, which warrants a higher level of risk acceptance [24,164,165].

Parents are faced with the challenging task of balancing the potential for a cure or improvement with the uncertainties of associated risks, often while navigating significant emotional stress. Research has indicated that parents often experience guilt regardless of their decision to consent or refuse, feeling remorse for either exposing their child to experimental risks or for denying a potential cure. Investigators need to convey risks clearly and honestly, including acknowledging when outcomes or rare side effects are uncertain [59,163]. Throughout a pediatric trial, it is essential to reassess the risk/benefit ratio continuously, as this must foreground developmental consequences. An intervention that improves disease control could still carry enduring neurodevelopmental tradeoffs, and investigators should communicate uncertainty transparently and revisit acceptability thresholds as new data emerge [77,158].

Another important ethical consideration is ensuring equitable access to pediatric nanotherapies. The high cost and advanced nature of these treatments often lead to important questions about fairness and equity in access. Without deliberate measures, advanced nanosystems can exacerbate disparities—trial access clustered in affluent regions; post-approval affordability limiting uptake. Practical steps include multi-site collaborative networks, subsidized travel and lodging, compassionate use pathways, and pricing/access planning before approval to reduce inequities between tertiary centers and under-resourced settings [44,58].

Because pre-approval pediatric trials are necessarily small and short, long-term safety must be shifted partly into the post-market period [55,58]. Regulators have established systems for monitoring, yet enhancing these frameworks specifically for nanotherapies in children remains a crucial priority. The FDA’s Office of Pediatric Therapeutics, for example, oversees required safety reviews that focus on pediatric populations for drugs and biologics following their approval. In addition, specialists recommend establishing pediatric safety registries tailored for recipients of nanomedicine [77,158]. These databases would serve as systematic records for healthcare providers, documenting the outcomes of children who have received a nanotherapeutic, tracking not only immediate side effects but also growth, neurodevelopment, and various long-term health parameters [61]. By collecting data from numerous patients over several years, these registries have the potential to offer valuable insights into nanodrugs’ pediatric-specific issues, such as nanoparticle deposition in growth plates or interactions with vaccines, which may be overlooked by general pharmacovigilance efforts that primarily focus on adults [62].

Another suggestion involves improving international cooperation in monitoring products after they enter the market [59]. The FDA and EMA can trigger label updates as signals arise; sponsors may be asked to conduct Phase IV pediatric studies or maintain long-term cohorts as conditions of approval. Importantly, global data pooling and a shared definition of “nanoparticle-related adverse event” will be essential, given the rarity of individual cases; regulatory bodies can enhance data quality. Furthermore, integrating new digital tools can enhance surveillance by using electronic health records and AI analytics to identify patterns, such as rising inflammatory markers or MRI changes in children receiving a nano-drug [55]. Early experience in other pediatric domains shows that sentinel systems and structured reporting can detect actionable signals. Thus, creating safety registries and longitudinal cohorts specifically for pediatric patients in the field of nanomedicine serves to safeguard these young individuals while simultaneously fostering public confidence [59,63].

Societal acceptability hinges on transparent communication and trust. Parents and clinicians are likely to be more open to adopting innovative nanotherapies if they are assured that a proactive system is monitoring for potential delayed risks and that any findings will be quickly communicated to inform practice. Parents must feel assured that the healthcare team and researchers are dedicated to prioritizing their child’s well-being. Involving parents and patients in the decision-making process while protecting against exploitation and inequality allows addressing the ethical challenges that arise when providing nanotherapeutics to vulnerable pediatric groups [55,58,61].

In summary, responsible pediatric nanoneurology demands: (1) developmentally grounded design and endpoints; (2) consent/assent processes that acknowledge uncertainty while respecting the child’s voice; (3) continuous, pediatric-focused safety surveillance; (4) equity-oriented access from trials through coverage; and (5) transparent communication practices. These elements, implemented together, can convert promising nano-enabled mechanisms into ethically sound, regulatorily credible, and socially acceptable therapies for children.

## 6. Converging Technologies: Synergies Shaping the Future

Pediatric nanoneurology is increasingly driven by a design–test–learn pipeline in which in silico tools, advanced in vitro models, and clinical data streams inform one another (Table 5). The incorporation of artificial intelligence (AI) with nanomedicine is facilitating novel methodologies in tailored therapy for pediatric patients, especially within neurology. AI-assisted nanoparticle design narrows the search space for size, charge, ligand density, and release kinetics. It can prioritize constructs with a higher probability of BBB penetration or lower immune clearance in age-stratified populations [166]. Moreover, through computer simulations that predict the distribution of a nanoparticle in a child’s brain and determine the effective dosage before administration to an actual patient, AI is augmenting real-time surveillance and adaptive treatment utilizing nanoparticles [58,167]. In addition, coupling “smart” nanoparticles incorporating biosensors that send feedback (e.g., measuring drug levels or neural signals) with AI enables predictive therapy, allowing adjustment of drug release or dosing schedules on the fly based on the child’s real-time data. Such an approach could greatly reduce trial and error in finding the right dose for each child as the treatment “learns” and personalizes itself over time [168,169,170,171]. This predictive power can guide clinical decision-making. In pediatric neurology, where conditions like brain tumors, epilepsy, or neurodevelopmental disorders vary widely from child to child, these AI-nanoparticle synergies are particularly valuable, facilitating the move towards precision medicine [167,172].

On the bench, brain organoids and microfluidic chips are breakthrough technologies that provide realistic, ethical, and scalable models for pediatric neuropathology [168]. Scientists have succeeded in creating organoids that exhibit features of pediatric brain tumors and neural disorders, effectively replicating how pediatric brain cancers grow and respond to treatments in the lab. Thus, researchers can then test nanoparticle drugs on relevant organoids to avoid the ethical and practical issues of experimenting directly on a young patient or an animal. In a precision medicine framework, one could even grow a personalized organoid from a specific child’s cells, then try multiple nanodrug options on it to see which works best, before giving that drug to the child [59,169]. Moreover, microfluidics amplifies the power of organoids by adding a level of physiological realism. Organoid-on-a-chip (e.g., brain-, tumor-, and BBB-on-chip) technology places tiny organoids into microfluidic devices with channels that simulate blood vessel or cerebrospinal fluid flow, perfusing them with nutrients and drugs under controlled flow conditions, in a similar fashion to a living brain [172]. Significantly, organoids-on-chips improve consistency and scalability in experiments and supply age-appropriate efficacy and neurotoxicity readouts without direct pediatric exposure, accelerating iteration on carriers and payloads. They also support longer-term cultures, allowing the observation of chronic effects of nanodrugs on brain tissue development [168,173]. Organoid systems also provide comprehensive post-exposure investigations, encompassing immunohistochemistry, single-cell RNA sequencing, and electrophysiological evaluations [169].

A complementary in silico patient layer—digital twins anchored in PBPK/Pop-PK —facilitates the simulation of nanomedicine activity within a specific child’s physiology. This approach shows significant potential for enhancing pediatric precision nanomedicine, integrating data about organ sizes and maturity, blood chemistry, immune system status, and disease specifics (e.g., tumor location, genetic mutations), essentially creating a personalized simulation platform [58]. By simulating age-dependent absorption, distribution (including nano-specific transport and clearance), and response, these models enable hypothesis-driven exploration of schedules (e.g., spacing for repeat dosing) and routes (intranasal, CED, IV ± FUS) before exposing children, and they can be updated as first-in-child data accrue [171,172,173,174,175]. The payoff of pediatric digital twins in nanomedicine would be immense. Clinicians could virtually test multiple therapy options (different nanoparticle drugs, or combinations of nano and traditional therapies) on the twin and identify which approach yields the best tumor shrinkage with the least toxicity. Moreover, digital twins can be continuously updated with real-world patient data, as the child grows or as their disease evolves; new data from scans or blood tests can recalibrate the model, keeping it an up-to-date predictor of what might happen next [12,176].

On the execution side, closed-loop “smart” nanosystems couple endogenous triggers (pH/enzymes/redox) with external control (e.g., MRI-guided FUS) for on-demand release and regional exposure. This synergy represents an attractive paradigm where narrow therapeutic indices and developing brains require precision [67,79,80,85]. In addition, theranostic hybrids (e.g., Gd-dendrimers, SPION-based agents) integrate imaging and therapy, enabling real-time response tracking and adaptive dosing, a particularly valuable feature in pediatrics where rapid readouts can spare non-responders unnecessary risk [86,140,177,178]. These platforms are not only mechanistically elegant, but they also directly mitigate the two main translational hurdles in children: uncertainty about where the drug goes and when it is acting.

Table 5 provides the key technological convergences driving advances in pediatric nanoneurology, together with their pediatric relevance, representative applications, and translational readiness.

**Table 5:** Key Technological Synergies Advancing Pediatric Nanoneurology.

Technological Convergence	Core Functionality	Pediatric Relevance	Typical Use Case/Examples	Readiness	Refs.
AI-Enhanced Nanoparticle Design	Optimize nanoparticle size/charge/ligands using predictive models and big data Predict BBB penetration, immune clearance, dosing	Improves targeting and safety profiles of treatments for developing nervous systems Reduces trial-and-error Adapts to age-dependent PK/BBB; safer starting doses	In silico screening of liposomal/polymeric variants Prediction of FUS parameters	Preclinical/tooling in early clinical planning	[41,179,180]
Organoids and Microfluidic Chips	Model pediatric brain disorders in vitro using 3D cultures and simulate nanomedicine interactions.	Provides ethical, reproducible, and age-appropriate efficacy/toxicity readouts without direct pediatric exposure	BBB-on-chip Glioma organoid penetration assays Seizure-like activity readouts	Preclinical (rapidly maturing)	[181,182,183]
Digital Twins and PBPK Systems	Create virtual patient-specific models to simulate nanotherapy outcomes and guide precision medicine.	Reduces trial-and-error in dosing and therapy design Increases safety and personalization.	Virtual trialing of nanoparticle regimens for DIPG/epilepsy Age-tier PBPK	Preclinical to planning; validation growing	[41,61,170,181,184,185,186]
Closed-Loop “Smart” Nano-Systems	Sensor-linked or triggerable carriers (pH/enzyme/redox) + external control (FUS)	On-demand release Potential seizure prevention Fewer invasive procedures	EEG-triggered release concepts MRI-guided FUS with co-infused nanodrugs	Preclinical/early feasibility	[8,67,72,76,79]
Theranostic Hybrids	Co-integrated imaging + therapy	Real-time response tracking Adaptive dosing in children	Gd-dendrimers, SPION-based probes for “see & treat”	Preclinical/early clinical imaging	[140,177,178]

## 7. Roadmap to Clinical Translation

The journey of pediatric nanomedicine from the confines of laboratory research to real-world clinical application faces several notable challenges. One issue is that nanoformulations that show stability at a small scale often face problems like aggregation or degradation when moving to larger-scale manufacturing processes [187,188]. The difficulties involved in scaling up are made even more complex by the need to comply with strict Good Manufacturing Practice (GMP) standards, since even small deviations can greatly affect nanoparticle characteristics. Maintaining consistency, sterility, and quality control across batches is essential during large-scale production. Implementing quality-by-design methodologies strategically, along with conducting early stability assessments, can help identify the optimal formulation conditions before scaling up the process [96,189,190]. Close collaboration between formulation scientists and process engineers is crucial. It should not be underestimated, as effective cooperation in multidisciplinary teams is key to refining nanoparticle design and manufacturing parameters concurrently.

A further challenge is implementing trials specifically tailored for pediatric populations. Recruiting and retaining children in clinical trials poses considerable challenges, largely stemming from the small patient populations and the ethical considerations involved [191]. To address this disparity, regulators and researchers have developed innovative strategies, such as including adolescents in adult Phase III trials when appropriate, creating study environments that meet children’s needs, incorporating age-appropriate information, and providing support for families [21].

Translational networks have emerged as a promising strategy. For example, the Institute for Advanced Clinical Trials for Children (I-ACT) consortium unites pediatric specialists and biopharmaceutical companies to design trials tailored specifically for children, thus expediting the development of innovative medications. These networks work alongside advocacy organizations to streamline the process of securing ethical approvals and share practical strategies for engaging pediatric participants. Research shows that partnerships among various sectors can greatly accelerate the progress of pediatric nanomedicine. A notable example is the Pacific Pediatric Neuro-Oncology Consortium (PNOC), founded in 2012 to improve treatment alternatives for children facing brain tumor diagnoses, which unites patients with specialized expertise and facilitates the execution of trials for conditions that are notably rare. The Children’s Brain Tumor Network (CBTN) emerged from a collaborative initiative involving both public and private sectors, aimed at promoting the sharing of data and biospecimens [192]. By openly sharing tumor samples and clinical data, CBTN provides researchers and companies involved in the development of nano-enabled diagnostics or therapeutics with valuable access. It is essential to recognize that PNOC and CBTN did not operate in isolation; instead, they created a collaborative translational ecosystem [21]. Philanthropic and industry partners are essential to this process, as the working groups actively engage with advocacy organizations for their insights. They also make use of funding from multiple institutions, including contributions from charitable foundations, to progress promising leads into clinical trials [193].

This initiative marks a significant step forward in the field of pediatric neuro-oncology nanomedicine. An important instance of collaboration is the FDA’s Pediatric Device Consortia (PDC) program. Although it focuses on devices, it illustrates how partnerships between academic institutions and industry can accelerate innovation. The creation of devices intended for pediatric use faces unique challenges, resulting in a historical trend of only a small selection of devices being designated explicitly for pediatric populations. To address this issue, the FDA provides funding to non-profit consortia that deliver seed grants and specialized services to innovators working in the pediatric sector [96,194]. Academic institutions are crucial for promoting scientific discovery and providing valuable clinical insights, while the industry contributes important expertise in development and helps create pathways to market. Government and philanthropic organizations often serve as a crucial unifying element by providing both funding and policy support [41].

The current market landscape presents a notable challenge because pediatric neurological disorders, especially the rare ones, represent a smaller and less financially appealing segment for pharmaceutical companies. Historically, this led to a significant challenge referred to as the “therapeutic orphan” issue, characterized by a lack of therapies specifically tailored for children. In light of this challenge, policymakers have introduced incentives designed to encourage research and development in pediatrics. The Orphan Drug Act of 1983, for instance, offers prolonged market exclusivity and tax benefits for therapies aimed at rare diseases [61]. Following the enactment of the Best Pharmaceuticals for Children Act (BPCA) in 2002 and the Pediatric Research Equity Act (PREA) in 2003, there has been a push to implement pediatric studies for newly developed medications, either through encouragement or mandates. The implementation of these measures has improved the overall landscape; however, they have not fully resolved the challenges, especially regarding pediatric neurologic conditions that often fall within the realm of rare diseases. To address this gap, Congress initiated the Rare Pediatric Disease Priority Review Voucher (PRV) program in 2012, which allows a company that obtains approval for a treatment targeting a serious, rare pediatric condition to receive a transferable voucher offering a faster review process by the FDA for another product [21].

Intellectual property and patents play a vital role in pediatric nanoneurology. Given the substantial costs associated with research and development, along with the unique attributes of nanotechnology products, robust intellectual property protection is essential for attracting investment [193], with recent trends showing a growing interest in patent activity in the areas of nanomedicine and nanobiotechnology [195,196,197]. This encompasses submissions that focus on pediatric applications, including nanoformulations of anticonvulsant drugs and nanocarriers intended for gene therapy aimed at genetic neurological disorders in children [21]. However, navigating the patent landscape can pose particular challenges. Nanotechnology often connects with various other fields (e.g., materials science, biology, medicine), leading patent examiners to require a clear demonstration of both novelty and utility, especially. To address this challenge, numerous academic technology transfer offices and companies are developing patent portfolios that integrate composition-of-matter claims for the nanomaterial alongside method-of-use claims aimed explicitly at pediatric applications [190]. This comprehensive intellectual property strategy increases the likelihood of securing substantial exclusivity that can be leveraged to form partnerships or licensing agreements, which are essential for acquiring the resources needed to progress a product through clinical trials. Alongside patents and vouchers, targeted funding programs and policy initiatives are enhancing the field. Numerous governments and international organizations have launched grant programs designed to support pediatric research that presents considerable risks alongside the possibility of substantial rewards [61,198].

The Pediatric Device Consortia grant program, established by the FDA in the United States, provides essential funding and expert guidance to initiatives focused on pediatric devices, including those in the field of neurotechnology. This support plays a crucial role in reducing risks linked to early development [61]. The National Institutes of Health (NIH) and the European Commission have dedicated resources to support pediatric translational research in neuroscience. This encompasses funding for pediatric neuroimaging tools provided by the BRAIN Initiative, as well as initiatives under EU Horizon Europe that focus on health technology specifically designed for children. Meanwhile, foundations focused on particular diseases like pediatric epilepsy, autism, and brain cancers are increasingly supporting nanotechnology approaches, recognizing their encouraging potential [194].

The integration of charitable donations, governmental assistance, and collaborative efforts from the industry can significantly accelerate initiatives that might encounter obstacles in a purely profit-oriented environment [188]. In the field of policy, regulators are crafting frameworks specifically designed for the pediatric use of nanomedicine. The FDA and the EMA have issued guidance concerning drugs that incorporate nanomaterials; nonetheless, this guidance often remains rather broad in scope. There is an increasing need for additional guidelines that consider the unique aspects of pediatric care [199,200]. This document provides recommendations for conducting juvenile animal studies aimed at evaluating the safety of nanodrugs. It also offers guidance on determining age-appropriate dosing of nanoparticles, considering that surface chemistries can interact in varied ways with developing physiological systems [193]. There is a clear advancement in the regulatory framework, with the EMA now requiring Pediatric Investigation Plans (PIPs) for new pharmaceuticals. This requirement highlights the significance of prioritizing pediatric formulations and clinical trials from an early stage, while also applying comparable mechanisms to advanced therapies and nanodrugs. Additionally, recent legislative efforts like the 2023 bipartisan Pediatric Rare Disease Cure Act in the United States seek to improve pediatric research by streamlining the ethics review process and providing research credits to companies focused on pediatric neurological disorders [198].

## 8. Conclusions

Nanotechnology is revolutionizing pediatric neurology by enabling solutions that were once thought to be beyond reach. This paper examined the potential of nanoscale systems in addressing ongoing challenges associated with the treatment of neurological disorders in children. Pediatric nanoneurology has evolved from proof-of-concept delivery vehicles to a convergent translational field that couples smart materials, precision imaging, and data-driven evidence generation. Across oncology, epilepsy/neuroinflammation, and neurodevelopmental and rare degenerative disorders, nano-platforms now target three complementary aims: (i) overcoming barriers, (ii) modulating biology, and (iii) measuring in real time. The use of nanoscale platforms has also progressed diagnostics, facilitating earlier disease identification, molecular stratification, and adaptive monitoring through recent advances like exosome-based “brain liquid biopsy,” ultrasensitive nano-biosensors, and targeted contrast agents that can spare children non-beneficial interventions. Furthermore, pioneering models like organoids and digital twins are enhancing the capacity to evaluate pediatric nanotherapies in silico or in vitro, thus diminishing dependence on invasive techniques and providing more tailored treatment approaches.

Nonetheless, the application of nanomedicine in clinical pediatrics must be approached with prudence. Certainly, as outlined in Section 4.6, most platforms examined in this analysis are still in the preclinical phase. Of those that progressed to clinical trials, many were discontinued or demonstrated only limited effectiveness. Extensive clinical implementation will require more substantial pediatric-specific trials, uniform safety and toxicity information, and regulatory structures designed for this demographic, rather than relying on extrapolated adult nanomedicine data. Developmental pharmacology and long-horizon safety require age-stratified design, hybrid endpoints, and post-market surveillance. Equally, ethics and equity must drive the use of these technologies, being currently viewed as intrinsic design constraints rather than add-ons. Ultimately, although nanotechnology presents unparalleled opportunities to transform pediatric health, its potential will only be realized through responsible innovation, thorough safety assessments, and inclusive policies that ensure children worldwide can benefit from these developments.

In summary, future progress is most likely to emerge from gains in precision (more localized exposure, faster and more informative response readouts, and dose minimization) rather than from singular curative breakthroughs. Altogether, targeted delivery platforms, sensitive diagnostic modalities, ethically and regulatorily aligned trial designs, and real-world evidence generation are beginning to convert previously intractable pediatric CNS problems into tractable, measurable, and progressively personalized interventions.

## Figures and Tables

**Table 1:** Key Pediatric-Specific Factors in Nanoneurology.

Consideration	Mechanism/Application	Pediatric Relevance	Solution/Implication for Nanocarriers	Refs.
BBB Development and Permeability	Tight junctions and transport systems mature across fetal → neonatal → infant → child stages.	BBB is largely functional at birth but undergoes postnatal refinement Transient vulnerability with inflammatory stressors	Timing delivery to periods of increased permeability; cautious exposure in early life to avoid unintended brain entry	[21,22,37,38]
Transporter ontogeny (PGP/ABCB1, BCRP/ABCG2)	Developmental shifts in efflux expression/activity at brain endothelium and choroid plexus	Age-dependent drug extrusion alters CNS exposure profiles	Surface ligands tuned to age-specific receptors; anticipate higher/lower efflux at different stages	[21,22,23,24]
Immune Response and Opsonization	Children’s immune systems are under development	Reduced opsonization/prolonged circulation vs. unpredictable immune sequelae Higher vulnerability to autoimmune-like responses	Nanoparticles can trigger exaggerated immune activation → necessity for biocompatible coatings and biodegradable nanocarrier materials	[31,32,34,39,40]
Neuroinflammation	Chronic inflammation is more damaging in immature brains Inflammation disrupts neural development	Potential long-term cognitive and behavioral effects	Anti-inflammatory payloads; avoid triggers that exacerbate microglial activation	[39,40]
Growth and Device Longevity	Rapid cranial/brain growth in early years	Implants may not scale with growth → mechanical stress/failure	Favor flexible, conformable electronics and bioresorbable interfaces Minimizes the need for repeated invasive procedures	[41,42]
Metabolic Enzyme Maturation (Hepatic Phase I/II)	Cytochromes and conjugation pathways are low at birth → mature over time	Altered biotransformation → risk of prolonged exposure/toxicity	Tune release kinetics Prefer degradable matrices Monitor active metabolite formation	[43,44,45]
Renal clearance maturation	GFR, tubular secretion/reabsorption is low in neonates → rise in infancy/childhood	Slower elimination and potential accumulation early in life	Adjust dose/intervals Track particle size/surface to enable renal/hepatobiliary clearance	[44,46,47,48]
Body composition and Vd	Higher total body water Lower fat in neonates/infants	Distribution differs for hydrophilic vs. lipophilic nanodrugs	Calibrate loading and hydrophilicity Expect age-dependent Vd shifts	[48]
Dosing Differences and PK strategy	Drug metabolism and distribution differ in children vs. adults Weight-based dosing + pediatric PK/PD variability	High inter-child variability High risk of under-/overdosing	Nanoparticles can alter drug absorption/distribution Need for pediatric-specific nanoparticle studies Use PBPK/Pop-PK modeling to select starting doses and titrate via TDM/response	[41,49]
Safety windows and long-term retention	Persistence of non-biodegradable materials; ABC phenomenon with repeat dosing	Unknown growth/organ effects with accumulation	Favor degradable/cleavable designs Plan spacing of repeat doses Monitor organ deposition	[50,51,52,53]
Consent and ethical considerations	Children cannot provide informed consent → responsibility falls on parents/guardians Clinical trials face stricter ethical oversight	Parental consent may reduce, but not remove, ethical risk Extra safeguards required for vulnerable populations	Tailored materials for families/child; consider independent advocates in high-risk trials Respect dissent when not life-saving; communicate uncertainties and alternatives	[39,41,54,55,56,57]
Post-trial surveillance	Pediatric safety registries Real-world evidence	Detect rare/late effects (growth, neurodevelopment)	Require long-term follow-up Harmonize international reporting	[58,59,60,61,62,63]

Abbreviations: BBB, blood–brain barrier; PGP, P-glycoprotein; BCRP, breast cancer resistance protein; Vd, volume of distribution; PBPK, physiologically based pharmacokinetic; Pop-PK, population pharmacokinetic; TDM, therapeutic drug monitoring.

**Table 2:** Innovations in Nanomaterials for Pediatric Neural Interfaces.

Concept	Application	Pediatric Relevance	Refs.
Graphene-Based Flexible Electrodes	Ultra-thin, highly conductive arrays Stable long-term neural recording/stimulation Flexible architecture reduces scarring High-resolution mapping of brain signals	Soft, conformable materials minimize irritation Safe for chronic use in developing brains Better tolerance in children with sensitive neural tissue Allows fine-scale monitoring during growth	[73]
Magnetoelectric Nanoparticles (MENPs)	Generate local electrical signals via magnetic fields Enable wireless, deep-brain stimulation Potential for minimally invasive neuromodulation Useful for seizure control or movement disorders	Non-invasive wireless control (reduces need for surgery) Suitable for very young children (<10 yrs) Lower infection risk compared to wired systems May help pediatric epilepsy or motor dysfunction	[21,73]
Conductive Polymer Nanocomposites (e.g., PEDOT Coatings)	Coatings reduce electrode impedance Improve recording/stimulation fidelity Increase the durability of implants Can incorporate drug-delivery functions	Low impedance enables gentle recording in neonates Enhances safety across pediatric age groups Supports long-term monitoring in children Minimizes risk of tissue damage over repeated use	[74]
MXene-Integrated Neural Interfaces	Highly conductive, MRI-compatible 2D materials Flexible for conformal brain contact Enhanced signal transduction Lightweight, thin electrodes	MRI-safe—important for repeated scans in children Flexibility reduces irritation during brain development Suitable for school-age and adolescent monitoring Supports imaging + therapy without radiation	[75]
Nano-Textured and Drug-Eluting Coatings	Nanopillars and grooves promote neuron adhesion Drug reservoirs allow controlled release Enhance biocompatibility of implants Reduce risk of inflammation or rejection	Encourage tissue integration in growing brains Aid healing after pediatric surgeries Localized drug delivery lowers systemic toxicity Helps maintain implant safety during development	[76]

**Table 4:** Clinical readiness of nanotherapeutic platforms discussed in this review (derived from Table 3).

Readiness Tier	Number of Platforms	Examples	References
Preclinical only	7 of 12 (58%)	PEG-gold NPs (glioblastoma); atomoxetine SLNs (ADHD); stem-cell exosomes (ASD); lamotrigine/phenytoin polymeric NPs (epilepsy); dendrimer/AuNP-lacosamide (epilepsy); enzyme/gene delivery (Batten/CLN2)	[77,112,118,126,127,136,138,140,141,142,144,145,146]
Early clinical (Phase I, ongoing/completed without major setback)	2 of 12 (17%)	MTX110 CED (DIPG, Phase I/ongoing follow-up); FUS + microbubbles (gliomas, early feasibility)	[50,85,89,109,112]
Early clinical, halted/terminated	3 of 12 (25%)	Liposomal doxorubicin (brain tumors, Phase I/halted); ferumoxytol imaging (terminated, supply issue); liposomal irinotecan (Phase I, no further progression reported)	[109]

## Data Availability

No new data were created or analyzed in this study. Data sharing is not applicable to this article.
